# Diverse mechanisms activate the PI 3-kinase/mTOR pathway in melanomas: implications for the use of PI 3-kinase inhibitors to overcome resistance to inhibitors of BRAF and MEK

**DOI:** 10.1186/s12885-021-07826-4

**Published:** 2021-02-06

**Authors:** Khanh B. Tran, Sharada Kolekar, Anower Jabed, Patrick Jaynes, Jen-Hsing Shih, Qian Wang, Jack U. Flanagan, Gordon W. Rewcastle, Bruce C. Baguley, Peter R. Shepherd

**Affiliations:** 1 Department of Molecular Medicine and Pathology, University of Auckland, Auckland, New Zealand; 2 Auckland Cancer Society Research Centre, University of Auckland, Auckland, New Zealand; 3 Maurice Wilkins Centre for Molecular Biodiscovery, Auckland, New Zealand

**Keywords:** mTOR, PI 3-kinase, PI3Kα, PI3Kδ, PIK3CA, PIK3CD, PIK3CG, A66, IC 87114, VPS34, Drug resistance, Melanoma, KU-0063794, BEZ-235

## Abstract

**Background:**

The PI 3-kinase (PI3K) pathway has been implicated as a target for melanoma therapy.

**Methods:**

Given the high degree of genetic heterogeneity in melanoma, we sought to understand the breadth of variation in PI3K signalling in the large NZM panel of early passage cell lines developed from metastatic melanomas.

**Results:**

We find the vast majority of lines show upregulation of this pathway, and this upregulation is achieved by a wide range of mechanisms. Expression of all class-IA PI3K isoforms was readily detected in these cell lines. A range of genetic changes in different components of the PI3K pathway was seen in different lines. Coding variants or amplification were identified in the *PIK3CA* gene, and amplification of the *PK3CG* gene was common. Deletions in the *PIK3R1* and *PIK3R2* regulatory subunits were also relatively common. Notably, no genetic variants were seen in the *PIK3CD* gene despite p110δ being expressed in many of the lines. Genetic variants were detected in a number of genes that encode phosphatases regulating the PI3K signalling, with reductions in copy number common in *PTEN*, *INPP4B*, *INPP5J*, *PHLLP1* and *PHLLP2* genes. While the pan-PI3K inhibitor ZSTK474 attenuated cell growth in all the lines tested, isoform-selective inhibition of p110α and p110δ inhibited cell growth in only a subset of the lines and the inhibition was only partial. This suggests that functional redundancy exists between PI3K isoforms. Furthermore, while ZSTK474 was initially effective in melanoma cells with induced resistance to vemurafenib, a subset of these cell lines concurrently developed partial resistance to PI3K inhibition. Importantly, mTOR-selective or mTOR/PI3K dual inhibitors effectively inhibited cell growth in all the lines, including those already resistant to BRAF inhibitors and ZSTK474.

**Conclusions:**

Overall, this indicates a high degree of diversity in the way the PI3K pathway is activated in different melanoma cell lines and that mTOR is the most effective point for targeting the growth via the PI3K pathway across all of these cell lines.

**Supplementary Information:**

The online version contains supplementary material available at 10.1186/s12885-021-07826-4.

## Background

Two major breakthroughs have occurred recently in melanoma therapy. The identification of common mutations in *BRAF* [1], *NRAS* [2] and *NF1* [3, 4] indicated that the RAF-MEK-ERK pathway is a crucial driver of melanoma. This has led to the development of drugs targeting mutant BRAF [5, 6] as a strategy for directly targeting melanoma growth. These targeted therapies can have impressive initial efficacy in tumours driven by *BRAF* V600 mutations. However, this is almost invariably followed by resistance development within a few months [7, 8]. More recently, therapies targeting immune checkpoints have delivered unprecedented therapeutic responses, but again there are groups of patients who do not respond or develop resistance to these treatments [9, 10].

One focus of the efforts to develop strategies to overcome this resistance to treatment has been on the PI 3-kinase (PI3K) pathway [7, 8]. This is because there is evidence some melanomas have inherent activation of the PI3K pathway [11], most notably through the finding that PTEN loss is seen in a subset of melanomas [12]. Activating mutations in *PIK3CA* are also found in some melanomas [13, 14]. Both PTEN loss and PIK3CA mutations have been shown to increase the rate of melanomagenesis induced by BRAF mutants in genetically-engineered mouse models [15, 16], which indicates the potential importance of these genetic alterations in melanoma biology. Signalling requiring the PI3K pathway and the activation of the PI3K pathway itself have both also been implicated in the development of melanoma’s resistance to BRAF/MEK inhibitors [13, 14, 17, 18] and immune checkpoint inhibitors [19, 20].

The above findings have lead to interests in understanding the role PI3K inhibition might play in overcoming therapeutic resistance in melanoma. Indeed, it has been found that inhibition of PI3K can potentiate the effects of BRAF and MEK inhibitors in attenuating the growth of melanoma cells [21] and melanoma tumours in both animal xenograft models [15, 21] and genetically-engineered mouse tumour models mentioned above [15, 16]. Furthermore, limited clinical response to a pan-PI3K isoform inhibitor was reported in a phase-1 clinical trial [22]. Notably though, the responses to PI3K inhibitors in these studies were highly variable, which highlights the need to understand how the PI3K pathway is regulated in different melanomas. However, a detailed evaluation of the full range of mechanisms by which the PI3K pathway is activated in melanoma is lacking. The high mutation burden and the genetic variability seen in melanoma [23] make it possible that a range of different genetic lesions might contribute to the activation of the PI3K pathway. Indeed, there is some evidence this might be the case [24] and, in line with this, mutations in *MTOR* [25] and several other genes of the PI3K signaling pathway [13, 26] have been identified in melanomas. Therefore, we undertook a study to comprehensively analyse the diversity of mechanisms that might contribute to the activation of the PI3K pathway in melanoma and to understand how that impacts on the type of PI3K pathway therapy that might best be used to treat melanoma. To study this, we took advantage of the large NZM panel of early passage melanoma cell lines, which we have shown to provide a good representation of metastatic melanoma’s genetic diversity and functional characteristics [27]. Our study used 75 of these cell lines and undertook genetic and biochemical analyses of components of the PI3K pathways. The results of these analyses were compared with responses to PI3K/mTOR inhibitors and their ability to overcome resistance to BRAF inhibitors. Evidence for PI3K pathway upregulation was found in virtually all of the NZM lines investigated, although the mechanisms by which this was achieved varied greatly, and it was found that targeting the PI3K pathway in the melanoma cells was most universally effective when done at the level of mTOR.

## Methods

### Cell lines

The cell lines used were chosen from a panel of NZM early passage cell lines that had been developed in house from biopsies of metastatic melanoma samples [27]. We first tested the effects of oxygen tension used for in vitro culture on drug response in a limited range of NZM lines and we found that the oxygen tension had an unpredictable effect on growth rates, expression of key signalling molecules and responses to drugs (Supplementary Fig. S1). Based on this, we chose to use the more physiologically relevant level of 5% O_2_ for our cell culture studies on the basis that this level was more likely to be representative of conditions found in a tumour. Cells were passaged in Minimum Essential Medium α (MEMα) supplemented with penicillin (100 U/mL), streptomycin (100 μg/mL), and amphotericin B (0.25 μg/mL; GIBCO Life Technologies), insulin (5 μg/mL), transferrin (5 μg/mL), and sodium selenite (5 ng/mL; Roche Diagnostics GmbH), and 5% fetal bovine serum (FBS).

### Genotyping

DNA was prepared from NZM cell lines and analysed using Sequenom mass array Oncocarta panels for analysis of mutations of common oncogenes [27]. Whole-exome sequencing was performed by the Liggins Institute Sequencing Service (University of Auckland, New Zealand) using an Ion Proton next-generation sequencing platform (Thermo Fisher) following the manufacturer’s protocol. Briefly, DNA were extracted and purified using a QIAamp® DNA Blood Mini Kit (Qiagen). Target regions were PCR amplified using a master mix composed of 100 ng DNA prepared in 5X Ion AmpliSeq HiFi Mix. The amplicons were partially digested by adding FuPa reagent (Thermo Fisher). Next, a mixture of two types of barcode adapters and DNA ligase were added to the pooled amplicons to generate an exome library, which was then loaded into an Ion PI™ Chip (Thermo Fisher), where each microwell received only one type of amplicons. Finally, the amplicons were sequenced by using an Ion Proton next-generation sequencing system. Raw reads were mapped to the human genome (hg38) and variant calling were performed using a Torrent Suite Software (Thermo Fisher). The criteria for filtering in copy number variants were both precision and confidence being larger than 10. The criteria for filtering in single nucleotide variants were allele coverage being more than 0.25 and minor allele frequency being less than 0.001, which we checked using values provided from the Ion Reporter software and the Genome Aggregation Database (gnomAD) of the Broad Instiute.

### Cell viability assay

Cells were seeded in 96-well plates (5000 cells/well) and after 24 h treated with inhibitors at a range of concentrations at which the drugs might be considered effective and selective. After 3 days of drug treatment, cell viability was determined using the sulforhodamine B (SRB) assay [28]. Results were expressed as percent of vehicle control from at least two independent experiments conducted in duplicate. GraphPad Prism software (GraphPad Software, version 8.0, San Diego, CA) was used to analyse the growth curves. A value of 100% inhibition is consistent with a cytostatic effect while values over 100% indicate cell death. The data points are plotted as the EC50 vs the max % inhibition and represent an average of 2–4 biological replicates, each done with two technical replicates.

### Western blotting

Western blotting was performed on lysates prepared from 75 NZM lines freely growing and harvested at 80–100% confluence. The protein isolation and immunoblotting procedures were performed as previously described from cells lysed in normal growth phase [29]. Enhanced chemiluminescence (Bio-Rad) was used to visualise the bands. Antibodies for immunoblotting were obtained from Cell Signaling Technologies, Millipore, and Sigma-Aldrich (listed in the appendix in Supplementary Fig. S2).

### Chemical reagents

Cell signaling inhibitors used were synthesized in house as previously described [30, 31] except for KU-0063794 and vemurafenib, which were obtained from Selleckchem and LC Laboratories.

## Results

### Correlations between genotypes and cell signalling pathway activation

**Fig. 1:**
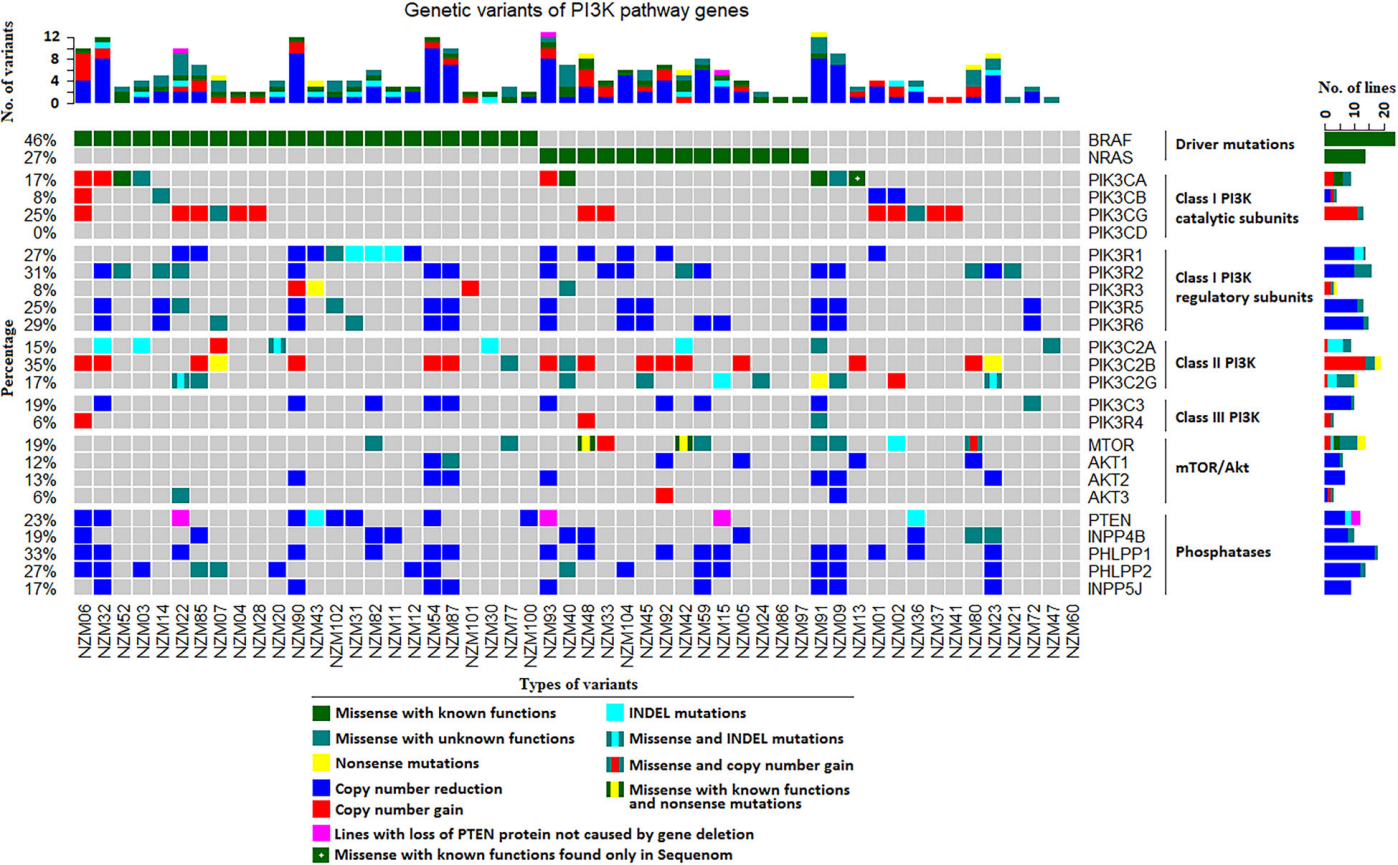
Genetic variants of the PI3K pathway genes. DNA extracted from NZM cell lines was subjected to whole-exome sequencing. The top row indicates the number of variants per cell line for the genes included. Middle rows indicate colour-coded individual variants found in 52 NZM cell lines. Side panels indicate the percentage of cell lines with mutations per gene. The bottom row indicates cell line names

The major genetic defects identified in the PI3K pathway in melanoma to date have been *PTEN* deletions and a small number of *PIK3CA* mutations. However, our genetic analysis of the NZM cell lines indicates a more complex picture of the PI3K pathway’s genetic alteration in the NZM cell lines, with many of the lines having variants in more than one gene in this pathway (Fig. 1). Of the 52 lines sequenced, 3 had *PIK3CA* oncogenic variants (NZM40 (H1047R) NZM52 (H1047R) and NZM91 (E545K)) validated by both Sequenom mass array and whole-exome sequencing (Supplementary Tables S1, S2). Another cell line had *PIK3CA* I391M variant, a variant previously identified in colon cancer but shown not to increase catalytic activity [32]. A *PIK3CA* variant at H1047L in NZM13 was seen only in Sequenom analysis. Amplifications in *PIK3CA* were also observed in 3 lines. We identified de novo coding variants in *PIK3CB* and *PIK3CG* in one and two lines, respectively (Supplementary Table S2). Surprisingly, there were no de novo genetic variants found in *PIK3CD* given that variants of *PIK3CD* have been identified previously in a range of cell lines of other cancer types [32]. The main novel findings for the class-IA catalytic subunits were that 11 of the lines have amplifications in *PIK3CG* and, in the case of the PI3K regulatory subunits (Supplementary Table S2), a number of lines showed reduced copy number and several lines also had de novo coding variants in *PIK3R1*, *PIK3R2*, *PIK3R3*, *PIK3R5*, and *PIK3R6* genes (Fig. 1 and Supplementary Table S3).

Copy number reductions of *PTEN* were found and as previously been reported in melanoma these were all in lines that also harboured *BRAF* mutations. We also identified common reductions in the copy number of other lipid phosphatases important in regulating the PI3K pathway. These include *INPP4B*, *PHLLP1*, *PHLLP2*, and *INPP5J*, with many lines having simultaneous copy number reductions in several of these genes as well as in *PIK3R* genes (Fig. 1, Supplementary Table S3, and Supplementary Table S4). Somewhat surprisingly, the genetic variants observed in the AKT genes tended to be copy number reductions rather than variants that would increase activity (Fig. 1). In the *MTOR* gene, 10/52 lines sequenced had de novo genetic variants (Fig. 1 and Supplementary Table S5). While the functional impact of these is not known, one novel missense variant was at the Ser2448 site which is a residue we have previously shown to be phosphorylated by Akt and to be involved in regulating mTOR activity [33]. This was found in 2 lines made for biopsies sequentially taken from the same patient. Western blotting of these lines (NZM42 and 48) confirmed the lack of phosphorylation of this site in these cells (Supplementary Fig. S1). These lines also harboured a Arg460Ter and Ile459Thr variants, a combination that was also seen in one other line. Further investigations into the mechanism by which these variants affect mTOR function are warranted.

Western blot analysis was performed on 75 of the NZM melanoma cell lines to understand expression levels of the PI3K signalling pathway components (Supplementary Fig. S1). This also is important for understanding whether epigenetic factors might be regulating the expression of genes such as *PTEN*. This revealed that the expression of all isoforms of class-1 PI3K could be found within the melanoma cell line panel. All the cell lines showed strong expression of p110α or p110β class-IA PI3K catalytic subunits. The majority of the cell lines also showed strong expression of, p110δ, the other class-IA PI3K isoform. This was not correlated with *BRAF*, *NRAS* or *TERT* genotype. Expression of p110δ is normally restricted to leukocytes but has been reported in melanocytes and melanoma [34] and a limited range of other cancers [35]. All cell lines expressed detectable levels of the class-IA PI3K adapter regulatory subunit p85α that is encoded by the *PIK3R1* gene (Supplementary Fig. S1). The majority of the cell lines also express the class-IB PI3K p110γ, an isoform with restricted tissue expression [36]. This isoform signals downstream of G-protein coupled receptors by engaging G-βγ subunits of heterotrimeric G-proteins in conjunction with its adapter subunit p101 (encoded by the *PIK3R5* gene). The majority of cell lines also express p101 indicating the signalling via class-IB PI3K is possible in these cells. Class II PI3Ks and Class-III PI3K were also detected in most of the cell lines (Supplementary Fig. S1, Supplementary Tables S6, and Supplementary Tables S7). Copy number reduction of *PTEN* [12, 37], *INPP5J* [38] and *INPP4B* [39, 40] have been reported to be relatively common in melanoma. These alterations are likely functionally important for PI3K signalling as they generally would downregulate the levels of the PI3K end-product PIP3. In total, 17 of the 75 cell lines exhibited loss of PTEN expression as measured by western blotting and 20 out of 75 cell lines showed absence of full-length INPP4B. The activity of the PIP3 effector Akt can be regulated by the PHLPP phosphatases [41]. Only 2 out of 75 cell lines showed a complete lack of full-length PHLPP1 and 6 out of 75 cell lines showed a complete lack of PHLPP2. No correlation was observed between low levels of PHLPP1 and the mutation status, although for PHLPP2, all but one of the low expressing lines were found to be *BRAF* mutant lines.

All lines exhibited strong expression of Akt1 and Akt2, which lie downstream of PI3K, even in the cell lines with copy number reduction of AKT genes (Supplementary Fig. S1). High-level expression of Akt3 has been reported in some melanomas and may contribute to vemurafenib resistance [42]. We find Akt3 expressed in 21 out of 75 NZM cell lines, although this does not correlate with BRAF or NRAS genotype. In addition, 30 out of 75 cell lines tested had high expression levels of pAkt - Ser473 while only 10 lines had high expression levels of pAkt-Thr308. The levels of pAkt in growing cells did not correlate with the Akt3 levels or with deletion status of *INPP4B* as has been previously reported [43, 44]. However, all the NZM lines with oncogenic *PIK3CA* mutations (3/75) or PTEN loss at the protein level (17/75) expressed high basal levels of pAkt-Ser473, which is in agreement with previous studies [30, 43].

### Understanding the role of PI3K in melanoma cell growth

**Fig. 2:**
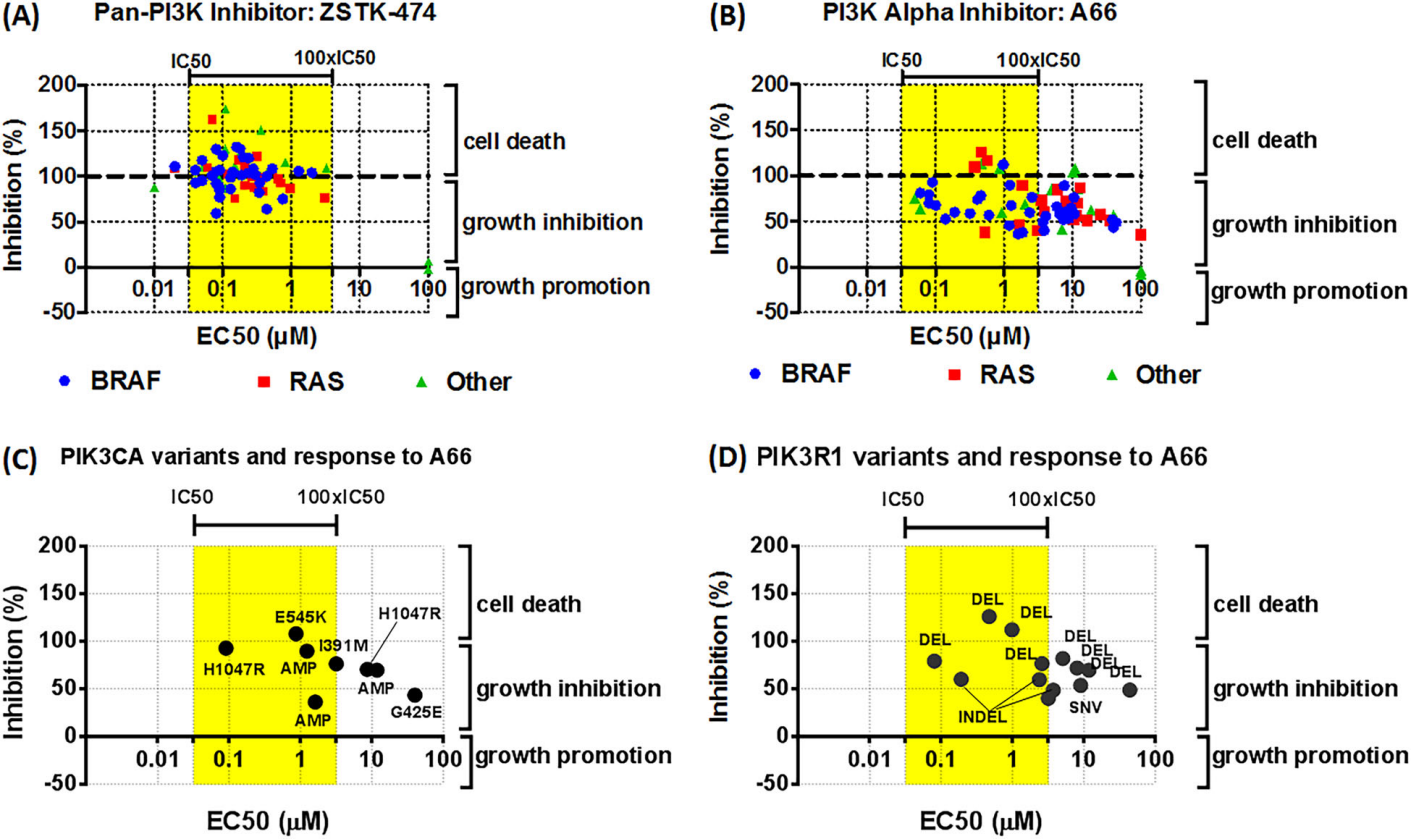
Role of PI3K in melanoma cell growth. Melanoma cells were seeded in 96-well plates (5000 cells/well) and treated 24 h later with inhibitors of (**a**) ZSTK-474), (**b**) A66, for 3 days. Cell viability was determined using the sulforhodamine B (SRB) assay. **c** Response to A66 in NZM lines with PIK3CA variants. **d** Response to A66 in NZM lines with PIK3R1 variants. BRAF: BRAF-mutant cell lines; RAS: RAS-mutant cell lines; other: cell lines wild-type for both BRAF and RAS. Data were from 2 to 4 independent experiments performed in duplicates. Yellow areas represent the concentration ranges between the biochemical IC50 and 100 x IC50 values of inhibitors for their specific targets; x-axis represents cell-based EC50 values; y-axis represents inhibition % at 100 μM of the corresponding inhibitors where 100% inhibition indicates complete stop of cell growth

**Fig. 3:**
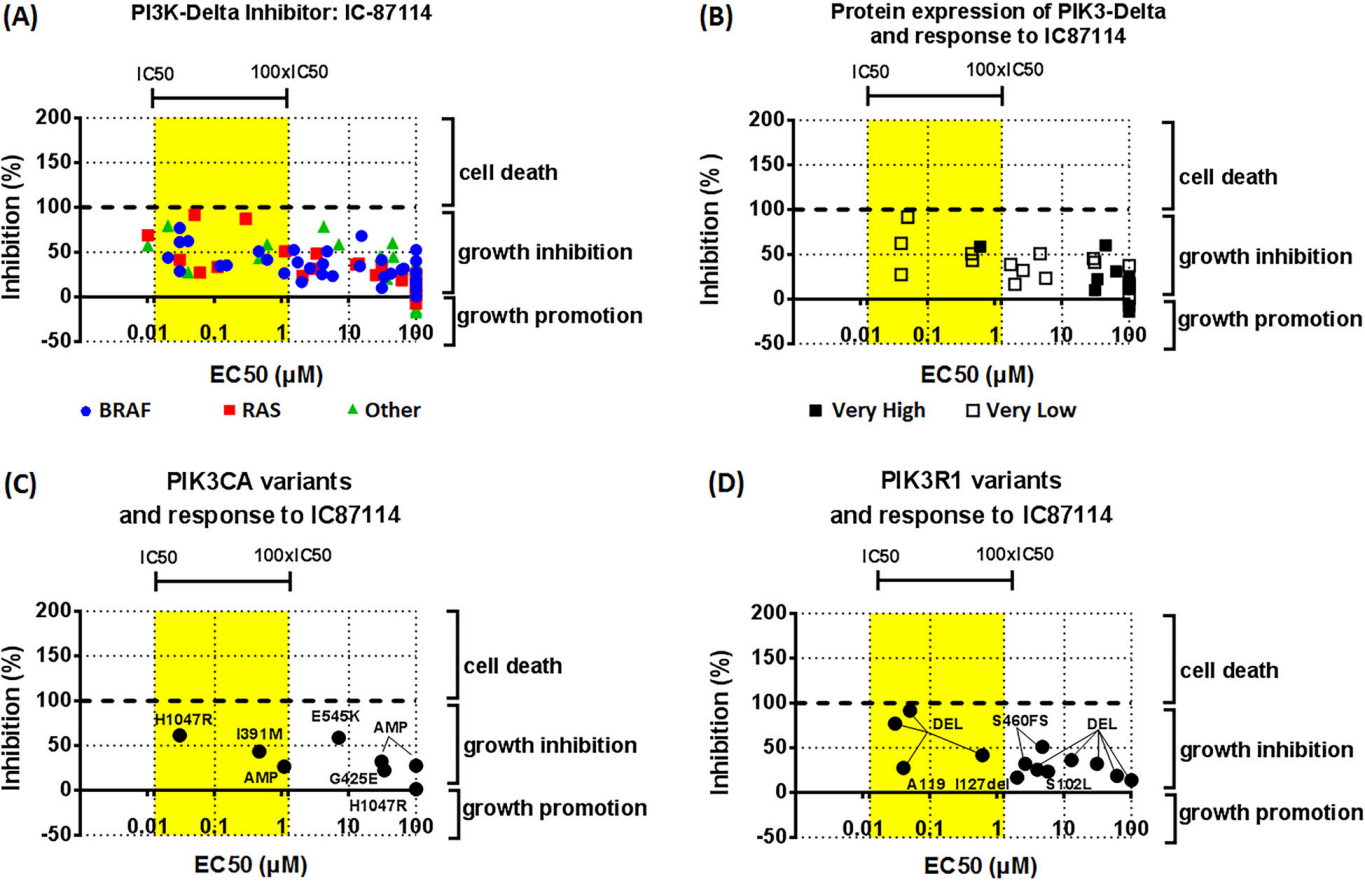
Role of the PI3-Delta isoform in melanoma cell growth. **a** Melanoma cells were seeded in 96-well plates (5000 cells/well) and treated 24 h later with inhibitors of IC87114. **b** Response to IC87114 in lines with high and low expression levels of PIK3-Delta isoform. **c** Response to IC87114 in lines with PIK3CA variants. **d** Response to IC87114 in lines with PIK3R1 variants. BRAF: BRAF-mutant cell lines; RAS: RAS-mutant cell lines; other: cell lines wild-type for both BRAF and RAS. Data were from 2 to 4 independent experiments performed in duplicates. Yellow areas represent the concentration ranges between the biochemical IC50 and 100 x IC50 values of inhibitors for their specific targets; x-axis represents cell-based EC50 values; y-axis represents inhibition % at 100 μM of the corresponding inhibitors where 100% inhibition indicates complete stop of cell growth

We next tested inhibitors of the PI3K pathway on cell growth in 5% oxygen in a larger panel of the cell lines (Supplementary Table S8). We first used ZSTK474, which is an nM inhibitor of all class-I PI3Ks but doesn’t inhibit mTOR at these concentrations [45, 46]. ZSTK474 strongly inhibited the proliferation of most NZM cell lines, irrespective of the driver mutation in these cell lines (Fig. 2a). However, this inhibition was largely through inducing cytostasis rather than causing cell death. Furthermore, we performed a Pearson correlation analysis and found no correlation between the response of NZM cell lines to ZSTK474 and their expression levels of AKT1, AKT2, AKT, and pAKT. Given the range of PI3K isoforms present, we next asked which of these was most important in the PI3K-mediated growth effects. However, isoform-selective PI3K inhibitors as single agents only attenuated growth of a subset of cell lines at concentrations at which these would be deemed to be acting in an on-target manner (Fig. 2b-e). The p110β inhibitor TGX221 [30, 47] and the p110γ inhibitor AS252424 [30, 48] had little effect on the growth of NZM cells (Supplementary Fig. S3). However, A66 [30, 49], a p110α selective inhibitor closely related to BYL719 attenuated the growth of a significant subset of cell lines in a concentration range up to 100 times the in vitro IC_50_ of the drug (Fig. 2b). The responsiveness of the lines appears independent of BRAF/NRAS status. The cell lines containing PIK3CA and PIK3R1 gene variants were not particularly sensitive to A66 (Fig. 2c, d). However, A66 tended to be less effective in lines showing loss of PTEN (Fig. 2e). The p110δ selective inhibitor IC-87114 [30, 50] attenuated growth in some cell lines in a concentration range up to 100 times the in vitro IC_50_ of the drug (Fig. 3a). Importantly though, growth inhibitory effects of A66 and IC87114 as single agents was much less than that of ZSTK474, which suggests that a drug targeting all class-I PI3Ks is effective in most of the NZM cell lines. Still, isoform-selective inhibitors had relatively little effect with only p110α and p110δ inhibitors having partial effects in a limited range of cell lines. Cell lines with high expression levels of p110δ tended to be more resistant to IC87114 (Fig. 3b). There was no correlation between PIK3CA or PIK3R1 status and response to A66 or IC87114 (Fig. 2 c, d and Fig. 3 c, d). Also, treating cells with a combination of A66 and IC87114 did not mimic the effect of the pan-PI3K inhibitor ZSTK474 (Supplementary Fig. S4).

### Efficacy of PI3K inhibitors in Vemurafenib resistant cell lines

**Fig. 4:**
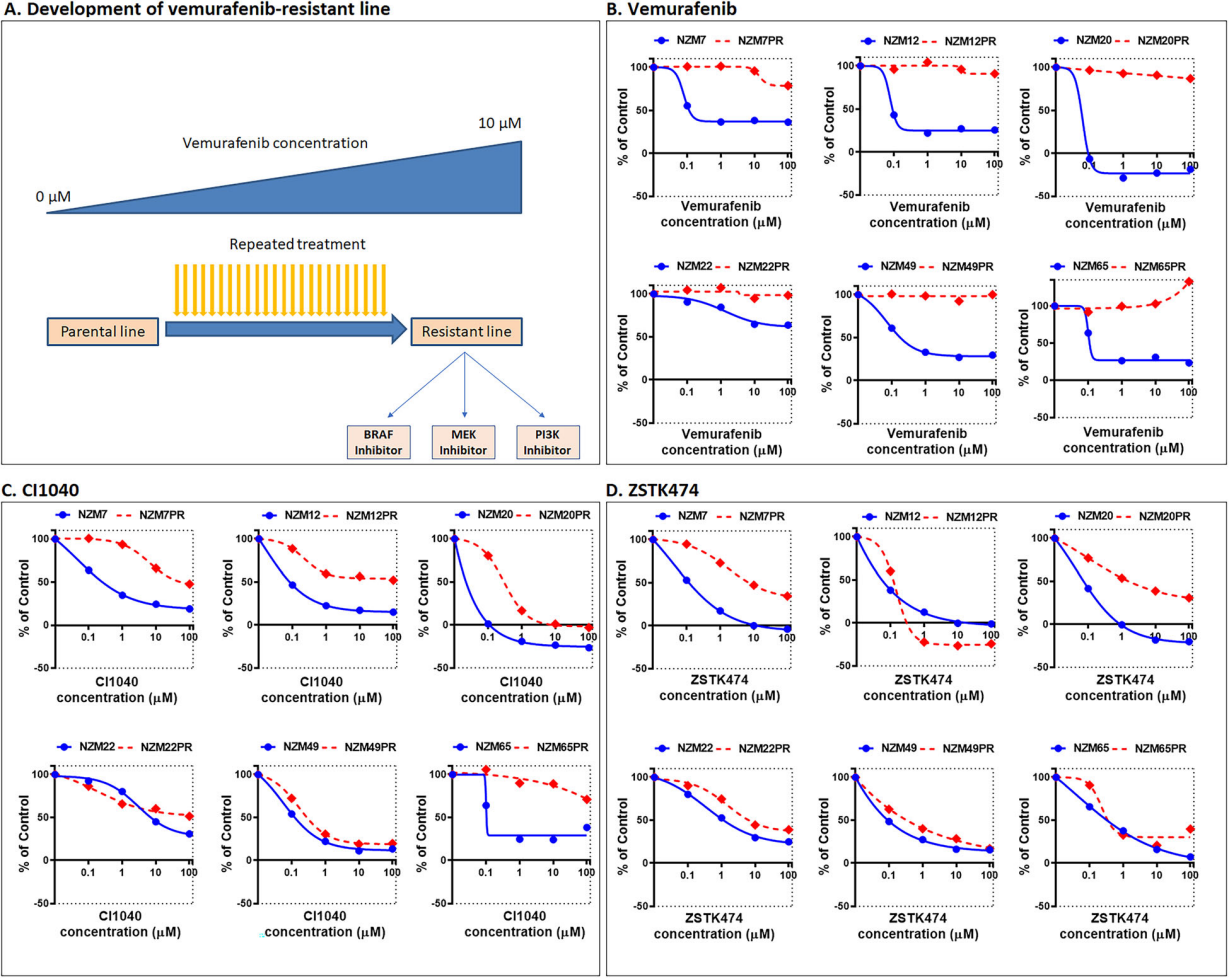
Efficacy of PI3K inhibitors in vemurafenib resistant cell lines. **a** Six melanoma lines, originally sensitive to vemurafenib, were treated with increasing concentrations of vemurafenib over a period of 2 months to make the drug resistant clones. These clones were then tested for their sensitivity for (**b**) Vemurafenib, (**c**) CI1040 and (**d**) ZSTK474. Cell viability was determined using the sulforhodamine B (SRB) assay. Data were from 3 to 4 independent experiments performed in duplicates

The findings that a pan-PI3K inhibitor is effective as a single agent in most NZM cell lines lead us to investigate whether it would potentially be effective in melanomas that had become resistant to BRAF inhibitors. For this, we took NZM lines that were initially sensitive to vemurafenib, CI1040 and ZSTK474 and cultured them in increasing doses of vemurafenib from 0.5 to 20 micromolar over a period of up to 3 months (Fig. 4a). Six of these lines developed vemurafenib resistance, these being named NZM 7PR,12PR,20PR,22PR,49PR and 65PR (Fig. 4b). This provided 6 model lines for studying identifying mechanisms for overcoming this resistance. In four of these lines, sensitivity to CI-1040 was also partially reduced (NZM 7PR, 12PR, 20PR and 65PR - Fig. 4b) showing that resistance to the MEK/ERK pathway can develop simultaneously with vemurafenib resistance. ZSTK474 remained effective in reducing cell growth in all of the vemurafenib resistant cell lines showing the potential for PI3K pathway inhibition in overcoming vemurafenib resistance. However, the sensitivity to ZSTK474 was partially reduced in 2 of those lines (Fig. 4c). This was accompanied by changes in the expression of some of the proteins involved in regulating PI3K activity (Supplementary Fig. S5). No evidence was found to suggest that changes in ERK signalling in resistant lines was impacting on the efficacy of PI3K inhibitors (Supplementary Figs. S5 and S6). This indicated that resistance to PI3K pathway inhibition could also occur.

**Fig. 5:**
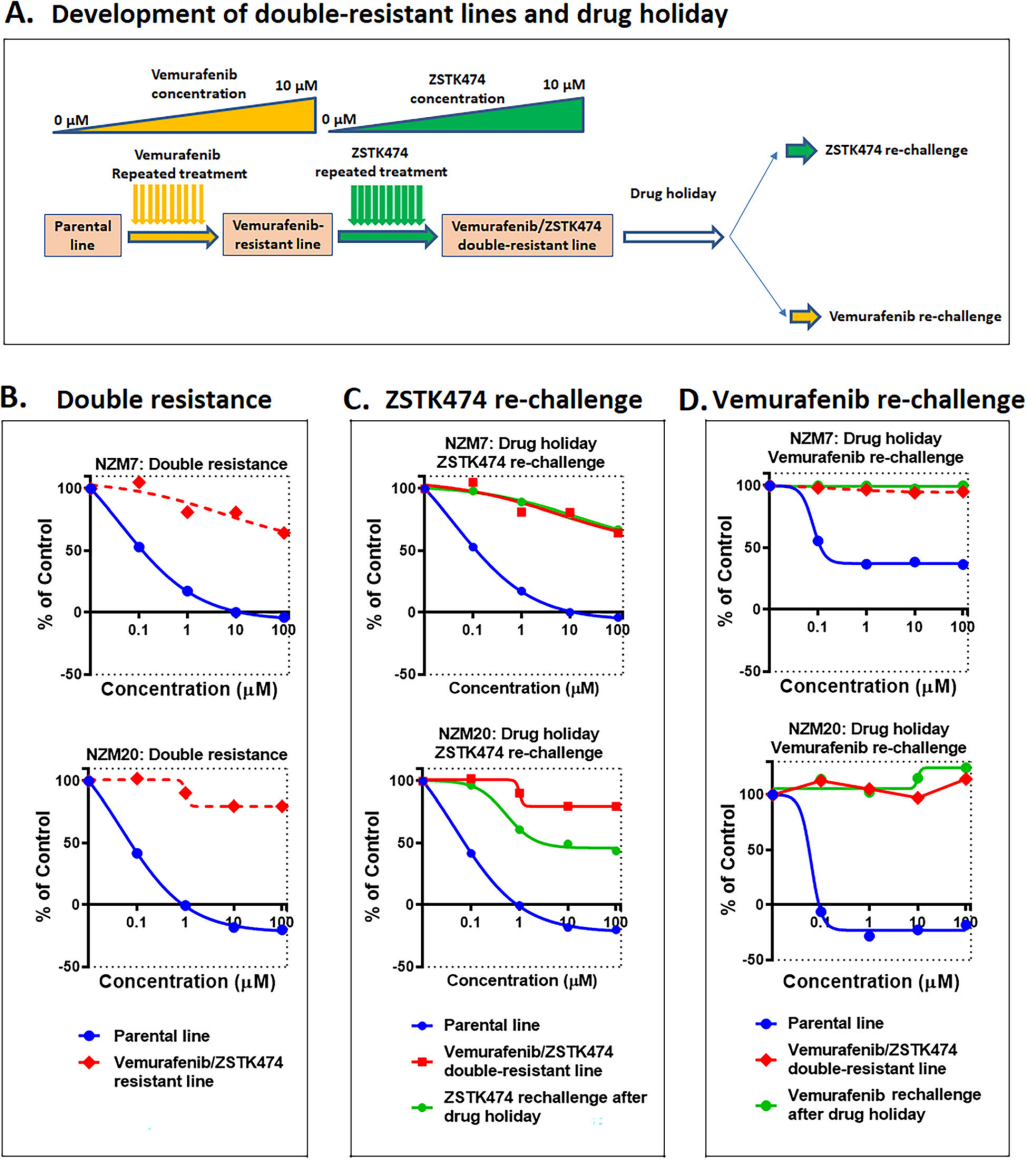
Efficacy of targeting PI3K in double-resistant cell lines. **a** Diagram of the development of double-resistant cell lines. NZM7PR and NZM20PR were maintained in increasing concentrations of ZSTK474 to create ZSTK474 resistant clones and tested for its sensitivity to the same as compared to its parental lines (**b**). Further the drug resistant clones were maintained in drug free media for at least 4 passages and re-challenged with drug in growth curve assay with ZSTK474 (**c**) and vemurafenib (**d**). All the data were a mean of 2 to 4 separate experiments carried out in duplicates. Data were from 3 to 4 independent experiments performed in duplicates

To explore the extent of the resistance to PI3K inhibition, we cultured these lines in increasing concentrations of the drug up to 10 μM to understand if full PI3K resistance could occur (Fig. 5a). This indeed developed in NZM7PR and NZM20PR, the two lines that had been partially resistant (Fig. 5b). We next asked whether this resistance to ZSTK474 was caused by a permanent change in the tumour cells, so we cultured the cells in drug-free media for at least 4 passages. Following this drug holiday, the cell lines were rechallenged with the drug during a cell growth assay. Interestingly, we find that one of the cell lines that were resistant to ZSTK474 regained some sensitivity to the drug (Fig. 5c) but that both cell lines remained resistant to vemurafenib (Fig. 5d), suggesting that both adaptive and acquired PI3K resistance can occur and that drug holiday might potentially be an approach to consider for resistance acquired to PI3K inhibitors in this context.

### Role of mTOR in cell proliferation

**Fig. 6:**
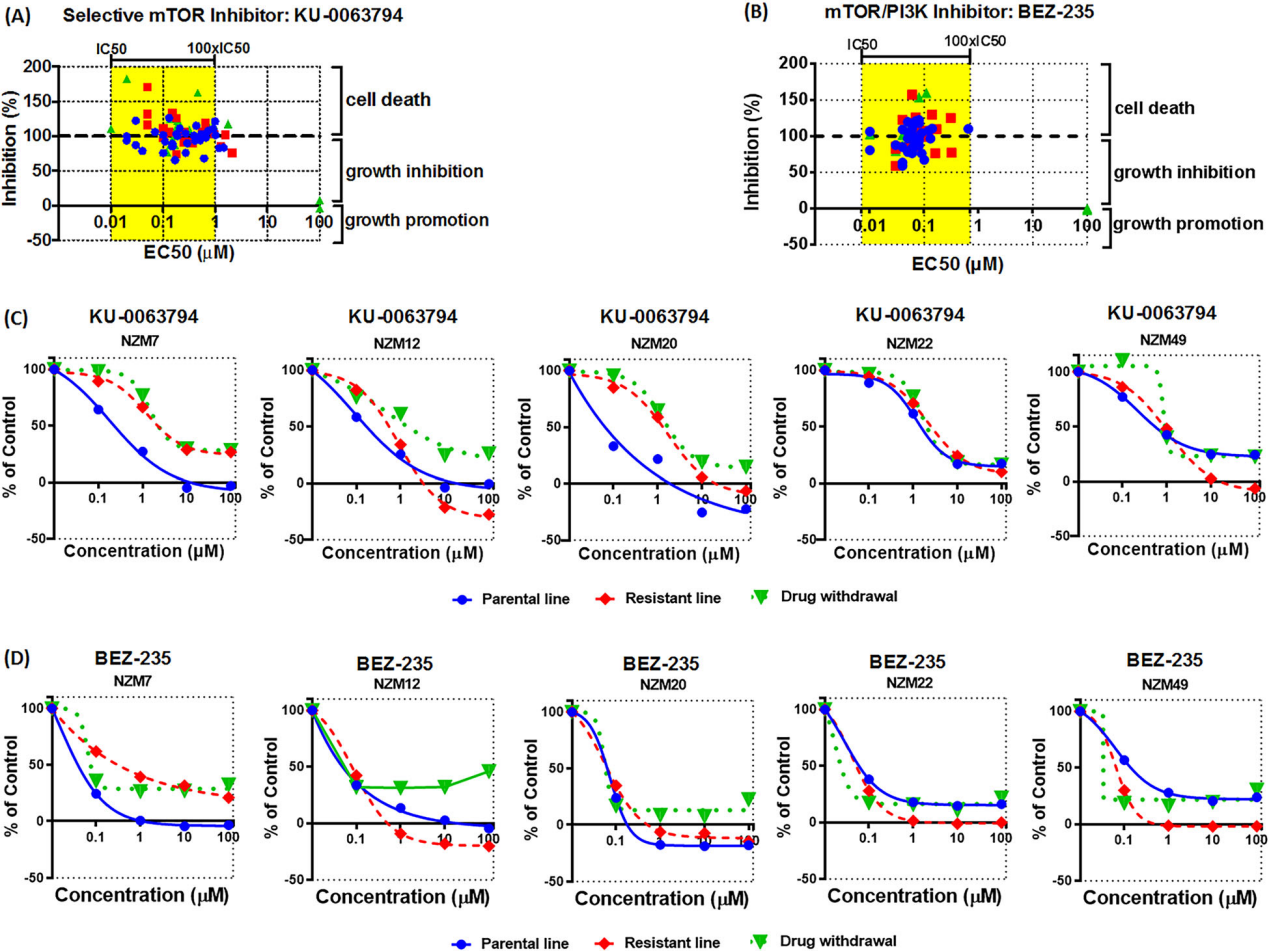
Role of mTOR in melanoma cell proliferation. Melanoma cells were seeded in 96-well plates (5000 cells/well) and treated 24 h later with inhibitors of (**a**) KU-0063794 and (**b**) NVP-BEZ235. Cell viability was determined using the sulforhodamine B (SRB) assay. **c**, **d** Five parental melanoma lines, their vemurafenib resistant (PR for NZM7, 12, 20, 22 and 49) and the respective drug withdrawn cells were tested for their sensitivity to mTOR inhibitor (**c**) KU0063794 and (**d**) mTOR plus PI3K dual inhibitor NVP-BEZ235. BRAF: BRAF-mutant cell lines; RAS: RAS-mutant cell lines; other: cell lines wild-type for both BRAF and RAS. Data were from 3 to 4 independent experiments performed in duplicates. Yellow areas represent the concentration ranges between the biochemical IC50 and 100 x IC50 values of inhibitors for their specific targets; x-axis represents cell-based EC50 values; y-axis represents inhibition % at 100 μM of the corresponding inhibitors where 100% inhibition indicates complete stop of cell growth

The finding that resistance developed to PI3K inhibitors, as well as vemurafenib, led us to investigate whether targeting mTOR might overcome these problems. To test the role of mTOR in cell proliferation, we used the selective mTOR inhibitor KU-0063794 [51]. These studies show that inhibition of mTOR gives a similar degree of inhibition in cells as that seen with ZSTK474, a drug that doesn’t cross-react with mTOR (Fig. 6a). Therefore we tested the effects of combining PI3K and mTOR inhibition to see whether there might be any synergistic effect. For this, we used NVP-BEZ235, a drug that is equally effective against mTOR and class-1 PI3K [52]. This was more effective at blocking cell growth than inhibiting either PI3K or mTOR alone across all 3 genotype groups (Fig. 6b). The EC_50_ of the effects was within a very tight range for all 75 cell lines consistent with it acting in an on-target manner. We next investigated whether mTOR inhibition might overcome the resistance that developed to BRAF and PI3K inhibitors. We found that these cells all remain responsive to mTOR inhibition (Fig. 6c and d).

## Discussion

This study represents the most comprehensive investigation to date of the relationship between genetic variation in the PI3K related oncogenes and tumour suppressor genes and responses to drugs targeting PI3K. This is possible because of the availability of a large number of NZM melanoma cell lines to understand responses to the diverse genetic changes in components of the PI3K pathway. We find that inhibition of all class-I PI3K isoforms effectively blocked the growth of the NZM cells. Previous studies in other cell lines have suggested that responses to selective inhibitors of PI3K isoforms might be linked to specific genetic alterations in the PI3Ks and related genes. For example, p110α inhibitors have been found to be more effective in some cell lines with *PIK3CA* mutations or amplifications [24, 30]. In addition, the effects of a p110β inhibitors have been seen to be more effective in PTEN deficient cells lines [53]. Previously before the whole-exome sequence data availability, we carried out a study using a small number of NZM lines, which showed none of the lines responded to single-agent treatment with isoform selective PI3K inhibitors [21]. Here we show that such responses do exist in some NZM cell lines, although the responses to p110α and p110δ inhibitors were only partial. However, these associations between genotype and drug response did not necessarily hold, and only some NZM lines with *PIK3CA* mutations or amplifications were very sensitive to A66, consistent with previous findings in melanoma lines [24]. Furthermore, selective inhibition of p110β was generally ineffective in attenuating the growth of NZM lines as a single agent, even in cells with *PTEN* loss. Another possible isoform-selective target is p110δ as the expression of p110δ in melanoma is unusual and its role is still not clear. The lack of genetic variants in *PIK3CD* (which encodes p110δ) is also notable. However, p110δ expression levels, in fact, correlated inversely with sensitivity to IC87114 in growth assays.

The lack of efficacy with individual isoform-selective inhibitors could be due to their effects being overridden by the dominant effects of other gene variants. This is unlikely to be the variants in *BRAF*, *NRAS* or the *TERT* promoter as there was no clear correlation between these and the response of PI3K inhibitors. It is though notable that 2 *PIK3CA* mutant lines resistant to A66 also have *KRAS* mutations. *PTEN* loss was, in fact, a better predictor of resistance to A66, which is consistent with previous studies in other tumour types where knockdown on *PIK3CA* did not affect PI3K signalling in several PTEN null cancer lines [54]. Together the results fit best with a model where multiple genetic variants in the PI3K pathway provide layers of functional redundancy in the class-1 PI3K system in melanoma as has been seen in some other cancer types [29, 30].

The findings above indicate that despite the increased risk of on-target toxicities, inhibitors targeting all class-IA PI3Ks would be required in strategies aimed at targeting the PI3K pathway in melanoma. Interestingly, although resistance to BRAF inhibition therapies can occur through different mechanisms such as reactivation of MAPK, PI3K, or TERT signalling, our data show that inhibition of all class-I PI3Ks significantly improves the efficacy of BRAF/MEK pathway inhibition, which is consistent with previous findings [24]. We induced BRAF inhibitor and MEK inhibitor resistance in a subset of the NZM cell lines. While the pan-PI3K inhibitor was effective in attenuating the growth of these cells, 2 of these lines developed a degree of resistance to ZSTK474, indicating that rapid development of resistance to PI3K inhibitors may be a limiting factor for the use of this form of therapy. This demonstrates that different melanomas are developing different resistance mechanisms and that, in some cases, the selective pressure caused by evading B-Raf inhibition is creating cells that are resistant to other drugs as well. This may explain why MEK inhibitors are clinically ineffective if used after B-Raf inhibitors fail [55]. We also find that further selective pressure with ZSTK474 caused partially resistant cells to develop a higher degree of resistance to the drug. This suggests that PI3K inhibitors also have the potential to fail if used in patients who develop resistance to B-Raf inhibitors. While this has not been extensively explored, a report of two patients treated in such a way found PI3K inhibition was ineffective [56]. It is also noteworthy to mention that one of our two PI3K resistant lines regained sensitivity to ZSTK474 after a drug holiday. This finding could be translational to clinical application, where patients developing resistance to PI3K inhibition therapies might be placed on a drug-free duration and subsequently treated again with the same therapies. Furthermore, mTOR lies downstream from PI3K, and both the vemurafenib- and ZSTK474-resistant cell lines remain sensitive to mTOR inhibitors. These provide a rationale for targeting mTOR to maximise the chance of avoiding the development of resistance to the drugs being used. Further rationale for targeting mTOR comes from recent findings that activating mutations in mTOR are relatively common in melanoma [57], something we also observe. To date, there are no reports of clinical trials of pure mTOR kinase domain inhibitors in melanoma, although in phase-1 trials of dual PI3K/mTOR inhibitors showed some clinical activity against melanoma [58]. These data suggest that mTOR inhibitors or mTOR/PI3K inhibitors may be more effective than PI3K inhibitors alone as part of combination therapy with BRAF and MEK inhibitors in treating melanoma.

## Conclusions

Our results indicate a high degree of diversity in the way the PI3K pathway is activated in different melanomas and that mTOR is the most effective point for targeting the growth via the PI3K pathway across all of these cells.

## Supplementary Information

**Additional file 1: Supplementary Table S1**: Hotspot mutations of genes relating to the PI3K pathway in NZM cell lines.

**Additional file 2: Supplementary Table S2**: Variants of genes encoding class I PI3K catalytic subunits

**Additional file 3: Supplementary Table S3**: Variants of genes encoding class I PI3K regulatory subunits

**Additional file 4: Supplementary Table S4**: Variants of genes encoding PI3K-related phosphatases

**Additional file 5: Supplementary Table S5**: Variants of genes encoding mTOR and AKT

**Additional file 6: Supplementary Table S6**: Variants of genes encoding class II PI3K

**Additional file 7: Supplementary Table S7**: Variants of genes encoding class III PI3K

**Additional file 8: Supplementary Table S8:** Summary of the major genotypes and drug response of NZM cell lines

**Additional file 9: Supplementary Fig. S1**: Effect of oxygen tension in drug response. Melanoma cells were cultured in either 5% or 20% oxygen tension. The cells were seeded in 96-well plates (5000 cells/well) and treated 24 h later with vemurafenib, CI1040, ZSTK474, A66, IC87114 and BEZ235. Cell viability was determined using the sulforhodamine B (SRB) assay.

**Additional file 10: Supplementary Fig. S2**: Expression at protein levels of PI3K pathway kinases in NZM cell lines.

**Additional file 11: Supplementary Fig. S3**: Role of PI3Kβ and PI3Kγ in melanoma cell growth. Melanoma cells were seeded in 96-well plates (5000 cells/well) and treated 24 h later with inhibitors of (A) TGX221 or (B) AS252524 for 3 days. Cell viability was determined using the sulforhodamine B (SRB) assay. BRAF: BRAF-mutant cell lines; RAS: RAS-mutant cell lines; other: cell lines wild-type for both BRAF and RAS. Data were from 2 to 4 independent experiments performed in duplicates. Yellow areas represent the concentration ranges between the biochemical IC50 and 100xIC50 values of inhibitors for their specific targets; x-axis represents cell-based EC50 values; y-axis represents inhibition % at 100 μM of the corresponding inhibitors where 100% inhibition indicates complete stop of cell growth.

**Additional file 12: Supplementary Fig. S4**: Effects of co-inhibition of the PI3Kα and PI3Kδ in melanoma cell growth. Melanoma cells were seeded in 96-well plates (5000 cells/well) and treated 24 h later with A66 alone, IC87114 alone, or combination of A66 and IC87114 for 3 days. Cell viability was determined using the sulforhodamine B (SRB) assay.

**Additional file 13: Supplementary Fig. S5**: Protein expression of key signalling molecules in 6 parental BRAF-mutant NZM cell lines originally sensitive to vemurafenib, along with the drug resistant clones of these lines. All the lines were grown in presence of complete growth media as described in the methods. (PR: vemurafenib-resisistant cell lines. PZR: vemurafenib/ZSTK474 double-resistant cell lines).

**Additional file 14: Supplementary Fig. S6**: The indicated NZM cell lines were seeded into 12-well plates (5 × 10^5^ cells per well) in serum-free medium for 24 h. The cells were stimulated with 100 nM insulin and then treated with 1 μM vemurafenib (Vem 1 μM), 1 μM ZSTK474 (ZSTK474 1 μM), 1 μM linsitinib (Linsitinib 1 μM), or 1 μM BMS754807 (BMS 1 μM). The positive controls (+ Control) for the experiment were cells that were stimulated with 100 nM insulin but not treated with any drug, and the negative controls (− Control) were cells that were not stimulated with insulin and not treated with any drugs. The lysates were western blotted with the indicated antibodies in duplicates

## Data Availability

Cell lines can be provided on reasonable request. Genomic data are available as described previously [27].

## Abbreviations

PI3K: Phosphoinositide 3-kinase
mTOR: Mammalian target of rapamycin
