# Supplementary figures and images for: Diverse mechanisms activate the PI 3-kinase/mTOR pathway in melanomas: implications for the use of PI 3-kinase inhibitors to overcome resistance to inhibitors of BRAF and MEK
Source: BMC Cancer. 2021 Feb 6;21:136. doi: 10.1186/s12885-021-07826-4 (PMC7866738; doi:10.1186/s12885-021-07826-4)

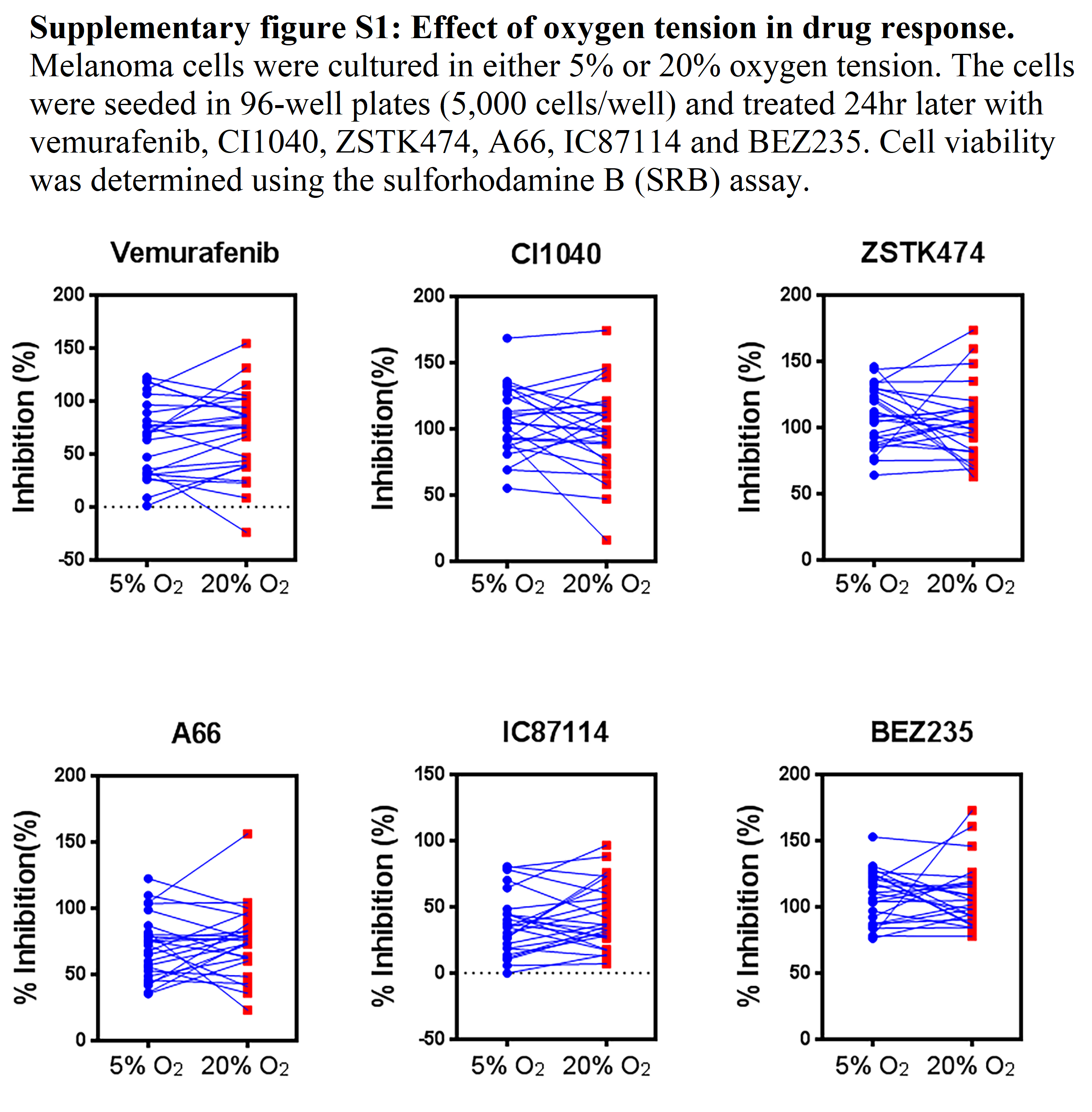

Supplement: Supplementary file 9 — Additional file 9: Supplementary Fig. S1: Effect of oxygen tension in drug response. Melanoma cells were cultured in either 5% or 20% oxygen tension. The cells were seeded in 96-well plates (5000 cells/well) and treated 24 h later with vemurafenib, CI1040, ZSTK474, A66, IC87114 and BEZ235. Cell viability was determined using the sulforhodamine B (SRB) assay. [file 12885_2021_7826_MOESM9_ESM.png]

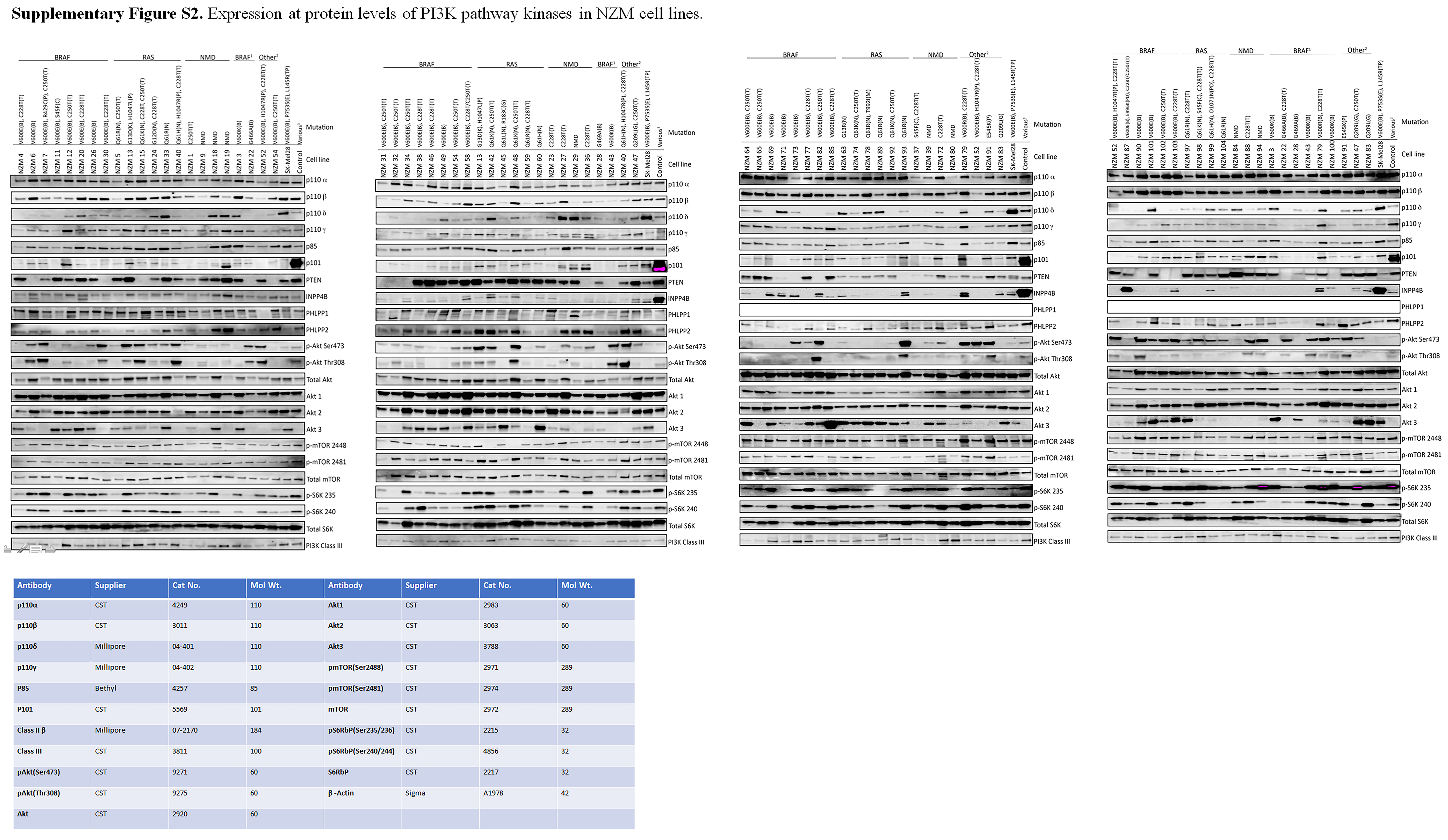

Supplement: Supplementary file 10 — Additional file 10: Supplementary Fig. S2: Expression at protein levels of PI3K pathway kinases in NZM cell lines. [file 12885_2021_7826_MOESM10_ESM.png]

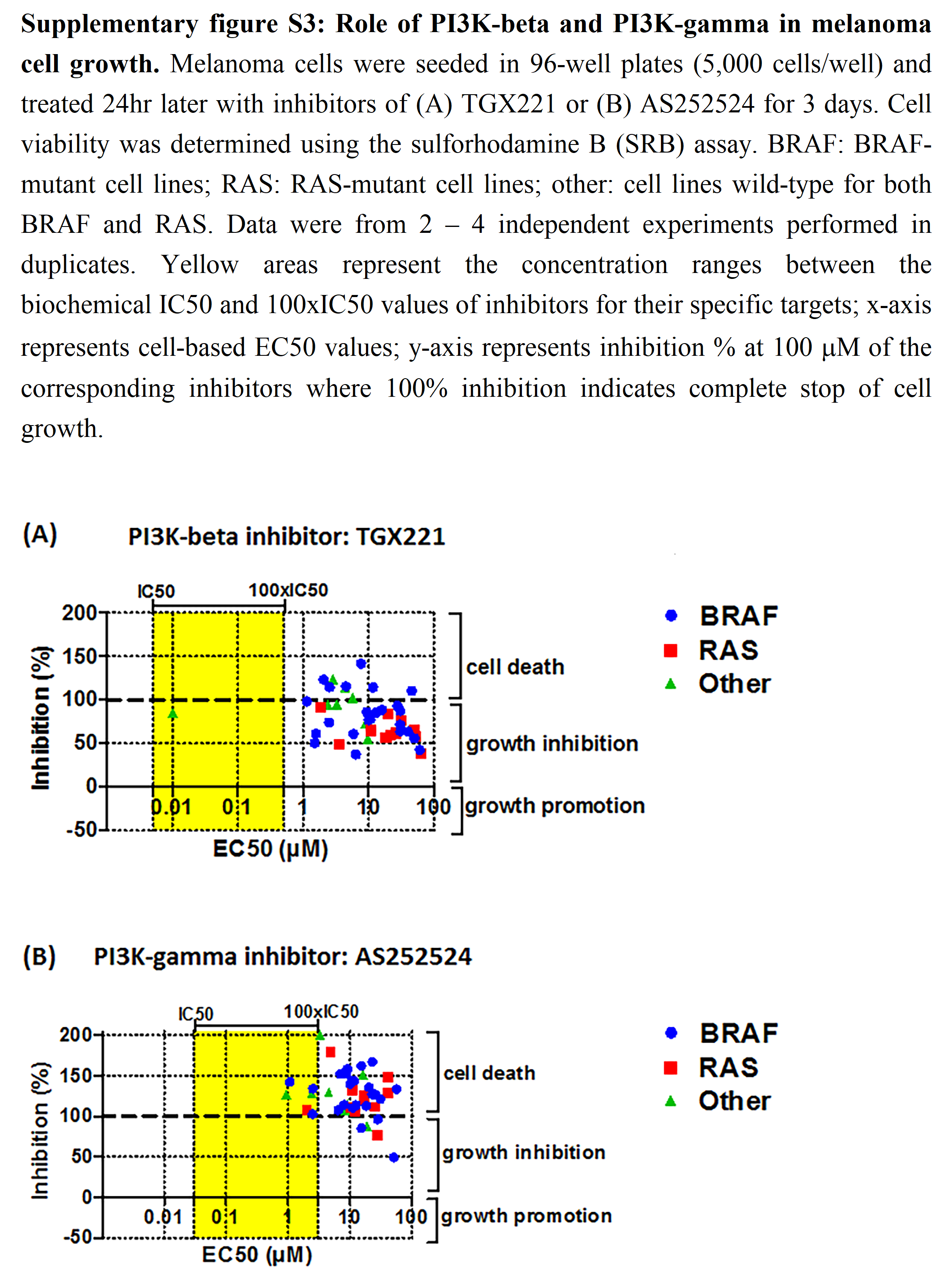

Supplement: Supplementary file 11 — Additional file 11: Supplementary Fig. S3: Role of PI3Kβ and PI3Kγ in melanoma cell growth. Melanoma cells were seeded in 96-well plates (5000 cells/well) and treated 24 h later with inhibitors of (A) TGX221 or (B) AS252524 for 3 days. Cell viability was determined using the sulforhodamine B (SRB) assay. BRAF: BRAF-mutant cell lines; RAS: RAS-mutant cell lines; other: cell lines wild-type for both BRAF and RAS. Data were from 2 to 4 independent experiments performed in duplicates. Yellow areas represent the concentration ranges between the biochemical IC50 and 100xIC50 values of inhibitors for their specific targets; x-axis represents cell-based EC50 values; y-axis represents inhibition % at 100 μM of the corresponding inhibitors where 100% inhibition indicates complete stop of cell growth. [file 12885_2021_7826_MOESM11_ESM.png]

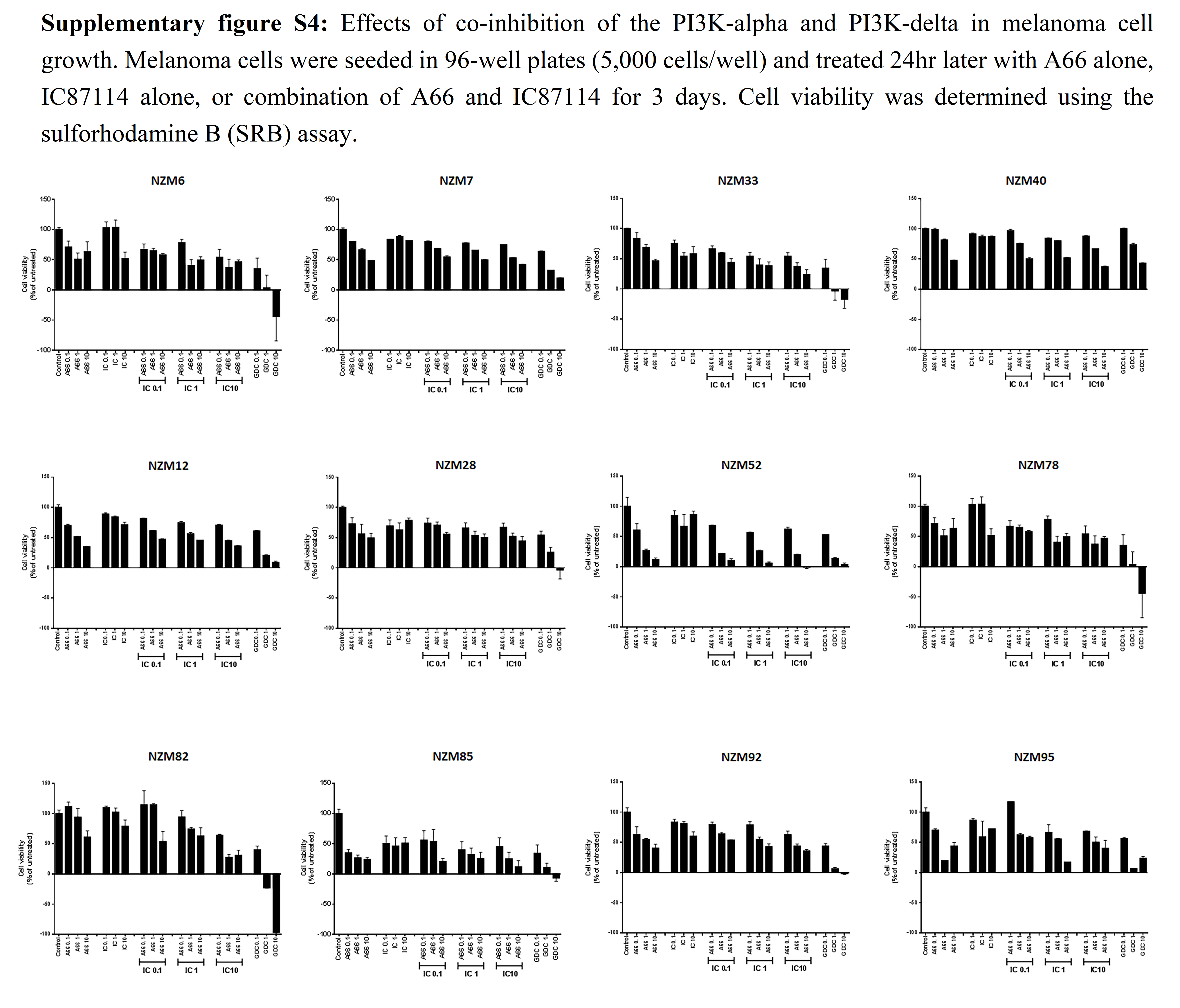

Supplement: Supplementary file 12 — Additional file 12: Supplementary Fig. S4: Effects of co-inhibition of the PI3Kα and PI3Kδ in melanoma cell growth. Melanoma cells were seeded in 96-well plates (5000 cells/well) and treated 24 h later with A66 alone, IC87114 alone, or combination of A66 and IC87114 for 3 days. Cell viability was determined using the sulforhodamine B (SRB) assay. [file 12885_2021_7826_MOESM12_ESM.png]

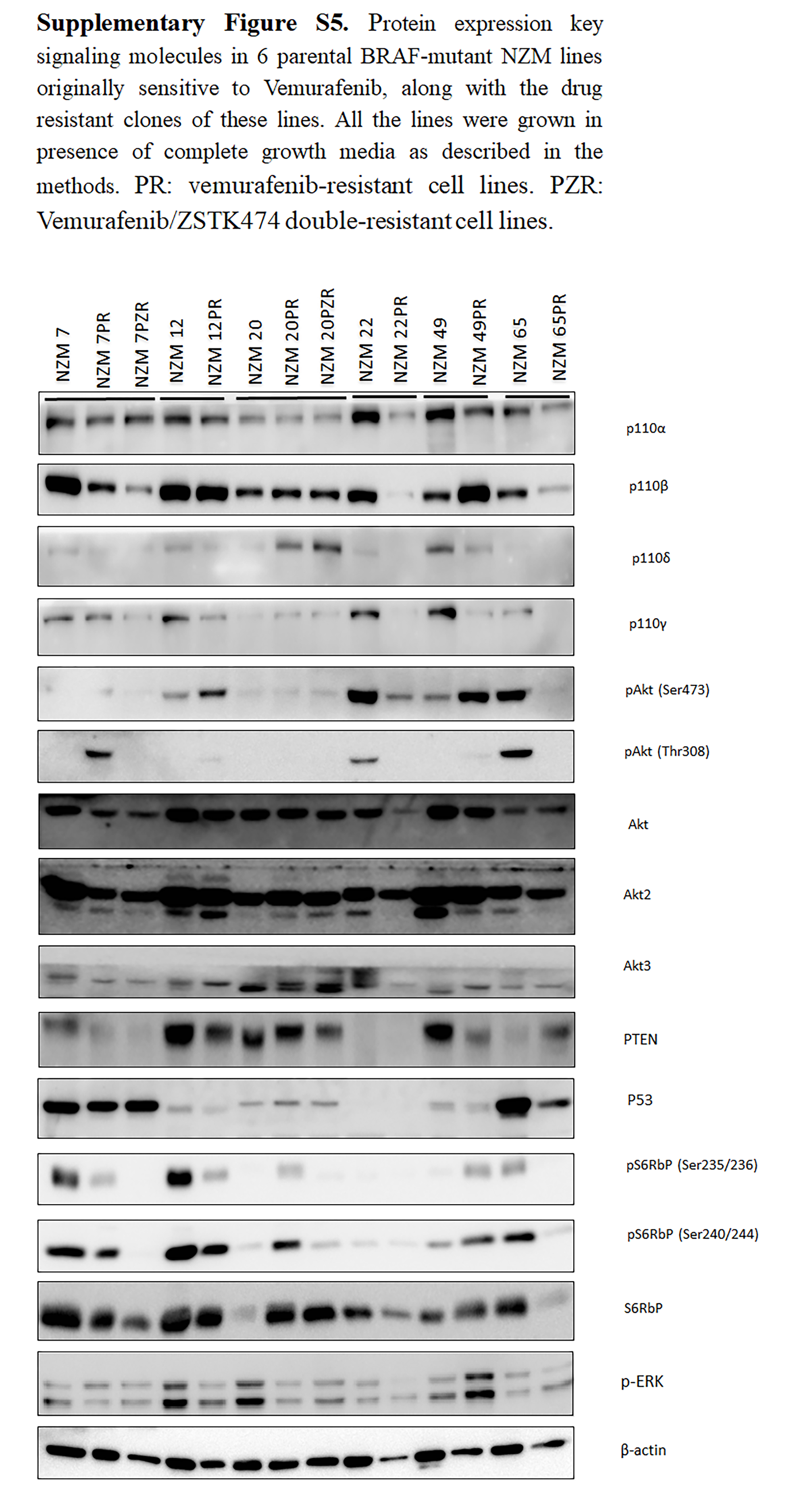

Supplement: Supplementary file 13 — Additional file 13: Supplementary Fig. S5: Protein expression of key signalling molecules in 6 parental BRAF-mutant NZM cell lines originally sensitive to vemurafenib, along with the drug resistant clones of these lines. All the lines were grown in presence of complete growth media as described in the methods. (PR: vemurafenib-resisistant cell lines. PZR: vemurafenib/ZSTK474 double-resistant cell lines). [file 12885_2021_7826_MOESM13_ESM.png]

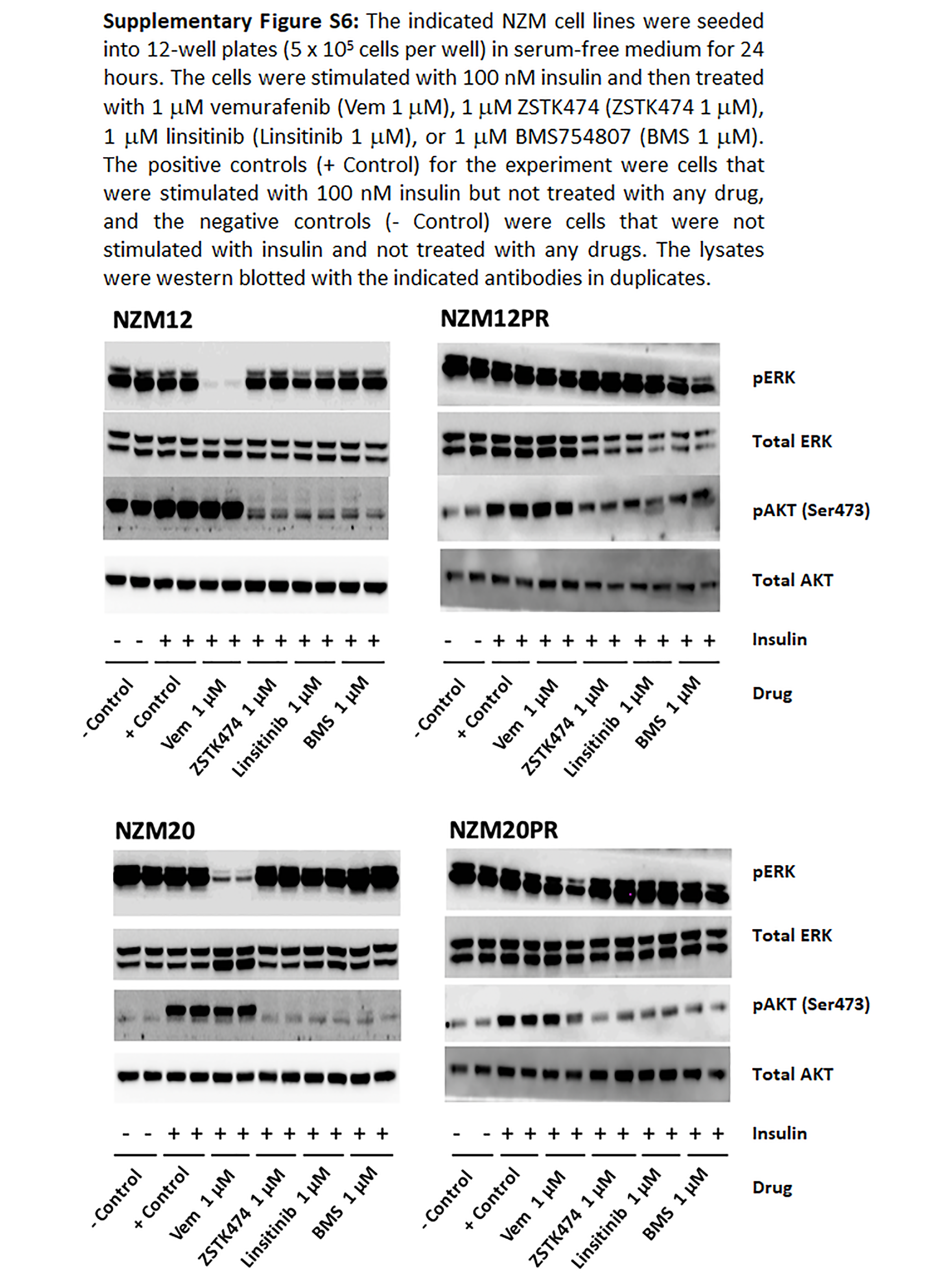

Supplement: Supplementary file 14 — Additional file 14: Supplementary Fig. S6: The indicated NZM cell lines were seeded into 12-well plates (5 × 10^5^ cells per well) in serum-free medium for 24 h. The cells were stimulated with 100 nM insulin and then treated with 1 μM vemurafenib (Vem 1 μM), 1 μM ZSTK474 (ZSTK474 1 μM), 1 μM linsitinib (Linsitinib 1 μM), or 1 μM BMS754807 (BMS 1 μM). The positive controls (+ Control) for the experiment were cells that were stimulated with 100 nM insulin but not treated with any drug, and the negative controls (− Control) were cells that were not stimulated with insulin and not treated with any drugs. The lysates were western blotted with the indicated antibodies in duplicates [file 12885_2021_7826_MOESM14_ESM.png]
